# Household air pollution from solid fuel use and depression among adults in rural China: evidence from the China Kadoorie Biobank data

**DOI:** 10.1186/s12889-023-16038-3

**Published:** 2023-06-06

**Authors:** Sek Ying Chair, Kai Chow Choi, Mei Sin Chong, Ting Liu, Wai Tong Chien

**Affiliations:** 1 The Nethersole School of Nursing, Faculty of Medicine, The Chinese University of Hong Kong, 6/F, Esther Lee Building, Horse Material Water, Shatin, New Territories, Hong Kong SAR, China; 2 School of Nursing, Sun Yat Sen University, Guangzhou, China

**Keywords:** Solid fuel, Cooking, Depression, Household air pollution

## Abstract

**Background:**

Solid fuels are still widely used for cooking in rural China, leading to various health implications. Yet, studies on household air pollution and its impact on depression remain scarce. Using baseline data from the China Kadoorie Biobank (CKB) study, we aimed to investigate the relationship between solid fuel use for cooking and depression among adults in rural China.

**Methods:**

Data on exposure to household air pollution from cooking with solid fuels were collected and the Chinese version of the World Health Organization Composite International Diagnostic Interview short-form (CIDI-SF) was used to evaluate the status of major depressive episode. Logistic regression analysis was performed to investigate the association between solid fuel use for cooking and depression.

**Results:**

Amongst 283,170 participants, 68% of them used solid fuels for cooking. A total of 2,171 (0.8%) participants reported of having a major depressive episode in the past 12 months. Adjusted analysis showed that participants who had exposure to solid fuels used for cooking for up to 20 years, more than 20 to 35 years, and more than 35 years were 1.09 (95% CI: 0.94–1.27), 1.18 (95% CI: 1.01–1.38), and 1.19 (95% CI: 1.01–1.40) times greater odds of having a major depressive episode, respectively, compared with those who had no previous exposure to solid fuels used for cooking.

**Conclusion:**

The findings highlight that longer exposure to solid fuels used for cooking would be associated with increased odds of major depressive episode. In spite of the uncertainty of causal relationship between them, using solid fuels for cooking can lead to undesirable household air pollution. Reducing the use of solid fuels for cooking by promoting the use of clean energy should be encouraged.

## Background

Depression is one of the most common mental health disorders, affecting more than 280 million people globally [1]. A recent systematic review and meta-analysis revealed that the 12-month and lifetime prevalence rates of major depressive disorder in China were 1.6% and 1.8%, respectively, and the percentages had been increasing over time [2]. If the population in China is estimated to be 1.426 billion in 2023 [3], the 12-month prevalence of major depressive disorder may reach over 22.8 million of individuals. A longitudinal population study in Australia suggested that the severity of depression is a major predictor for suicidal ideation and suicidal attempt [4]. Based on a recent meta-analysis on 15 studies, the prevalence of suicidal attempt in a lifetime among individuals with major depressive disorder was 3.45 times higher than those without major depressive disorder [5]. A study conducted in mainland China reported that the prevalence of suicidal ideation was 16.7% among 1,916 patients (18–70 years old) with major depressive disorder [6]. Symptoms of major depressive disorder, such as low mood, anhedonia and impaired cognition, are one of the key contributors to functional impairment [7], which could cause a great economic burden to the society. A study by Rayner et al. [8] reported that there was a significant correlation between total healthcare costs (i.e., accident and emergency department visits, hospitalizations, and visits to doctor) and depression. In addition, patients with multimorbidity and depression had more than twice the inpatient costs compared with those without depression [9]. The estimated health burden from depression has been continuously increasing over the years. More recently, a systematic review reported that major depressive disorder was accounted for 49 million disability-adjusted life-years in 2020 [10].

Approximately 2.4 billion people still use solid fuels such as animal dung, wood, and coal for cooking globally [11]. To date, solid fuels are extensively used in China, especially in rural households. There are half a billion people (40% of total population) in mainland China living in rural areas [12], with more than three-fourths of these rural households using solid fuels for cooking [13]. Incomplete combustion of solid fuels produces compounds such as carbon monoxide, sulphur dioxide, black carbon and PM_2.5_(fine particulate matter with a diameter of less or equal to 2.5 μm) [14]. Concentrations of PM_2.5_ from household air pollution due to cooking with solid fuels can be substantially high, causing up to 40% higher PM_2.5_exposure compared with the indoor and outdoor environments [15]. Previous studies have confirmed that the exposure to solid fuel use contributes to adverse health effects such as sleep disturbance [16], chronic bronchitis and obstructive pulmonary disease [17], hypertension [18] and an increased risk of cardiovascular disease hospitalization and stroke among rural population [19]. A nationwide prospective cohort study also reported significant association between using solid fuels for cooking and cardiovascular mortality in China [20].

A longitudinal study conducted in the United States reported that individuals with previous 30-day exposure to ambient fine PM were 1.2 times more likely to have moderate to severe depressive symptoms [21]. Based on a national longitudinal survey in China, cooking with solid fuels was associated with a higher risk of depressive symptoms among individuals aged 60 years and above [22]. Similarly, a cohort study also reported positive association between solid fuel use and depression [21]. However, the study conducted by Pun et al. [21] in the urban areas of the United States did not focus specifically on household use of solid fuels as the source of air pollution. Meanwhile, the studies conducted by Li et al. [22] and Shao et al. [23] were restricted to middle-aged and older Chinese population living in urban or rural areas.

Air pollutant emissions from solid fuels are associated with adverse health effects. In recent decades, improved cookstoves and combustion technologies have been implemented but a large number of individuals remain using solid fuels for cooking. Despite the important role of solid fuels in producing energy, its potential detrimental impact on mental health demands urgent attention. There is a lack of studies focusing on the use of epidemiological data to investigate the association between solid fuel use and depression in developing countries; concerns arise as China is the second most populous country where 40% of the population are living in rural areas and actively using solid fuels for cooking. It is crucial to investigate the relationship between these two variables specifically in rural China with a large-scale study. Therefore, this current study aimed to investigate the association between using solid fuels for cooking and depression in rural China.

## Methods

### Study design and population

This study employed a secondary data analysis using the baseline data from the China Kadoorie Biobank (CKB) study. The original CKB study was conducted between 2004 and 2008, which recruited over 0.5 million adults from 10 regions across mainland China. After providing informed written consent, each participant attended a face-to-face interview and a physical examination. A total of 512,681 adults aged between 30 and 79 years (without any major disability) with permanent residence were included in this baseline survey. A standardized electronic questionnaire was used to collect participant information including sociodemographic characteristics, lifestyle habits, exposure to passive smoking and domestic indoor air pollution, medical history, physical activity, and mental health status. The questionnaire used can be accessed via the official website of CKB (https://www.ckbiobank.org/study-resources/survey-data). Each participant’s resting blood pressure (BP) was measured using the A&D digital BP monitor (Model No.: UA-779). A body composition analyzer (Model No.: TBF-300GS) was used to measure body mass index (BMI), while a standing height measuring instrument was used to measure weight and height. BMI is calculated by using this formula: the participant’s weight in kilograms (kg) divided by the square of height (H) in meters (m), (BMI = kg/ H^2^) [24]. BMI at 28 kg / m^2^is recommended as the cut-off point for obesity for the Chinese people [25, 26]. More detailed information about the original CKB study has been previously reported [27–29]. Ethics approvals for the CKB study were obtained from the Chinese Center for Disease Control and Prevention (Approval Notice 005/2004) and the Oxford Tropical Research Ethics Committee (OxTREC Ref: 025–04) of the University of Oxford. The study was conducted in line with the principles outlined in the Declaration of Helsinki.

### Measurements

#### Exposure to household use of solid fuels

The approach of Yu et al. [20] was followed to calculate the durations of exposure to solid fuels used for cooking and heating separately. Participants were asked to provide detailed information about their exposure to household use of solid fuels for cooking and heating, including related information such as the duration (in years) they lived in their three most recent residences, frequency of cooking in each residence, types of fuels used for cooking and heating, and availability of cookstove ventilation (chimney or extractor). Participants who reported that they cooked less often than once a month in a residence were considered as noncooking and regarded as having no exposure to solid fuel used for cooking. Participants who reported that they cooked at least once a month were then asked to provide additional information related to the types of primary fuels they used. There are two categories of primary fuels, namely “clean fuels” such as gas and electricity, and “solid fuels” such as wood and coal [30]. The total duration (in years) of household use of solid fuels for cooking was calculated by summing up the duration of using solid fuels as the primary cooking fuel in each residence. Likewise, participants who used solid fuels for heating in winter were asked further questions about the types of primary fuels they used, and the total duration (in years) of household use of solid fuels for heating was calculated by summing up the corresponding duration in each residence. The level of exposure to solid fuels used for heating was estimated by multiplying a weight coefficient to years of solid fuels used for heating, of which the weight coefficient was calculated based on the average portion of years with temperature less than 8 degree Celsius in each of the residences from 1999 to 2013, ranging from 0.18 to 0.42, as detailed in Yu et al. [20].

### Depression

In this study, major depressive episode was evaluated by the Chinese version of the World Health Organization Composite International Diagnostic Interview short-form (CIDI-SF) [31]. As there is no gold standard for assessing mental disorders in the CIDI-SF, this version was calibrated rather than validated and produced similar population estimates of major depressive episode to the Structured Clinical Interview for DSM-IV, which is a state-of-the-art clinical research diagnostic interview tool for mental disorders [32]. Participants were first asked whether they had any of the following symptoms lasting for ≥ 2 weeks in the past 12 months: a) feeling much saddened, or depressed than usual; b) loss of interest in most things like hobbies or activities that usually gave you pleasure; c) feeling so hopeless and loss of appetite even for your favorite food; d) feeling worthless and useless, everything that went wrong was your fault, and life was very difficult with no way out. If participants answered “yes” to any of the above-mentioned situations, they were further assessed for major depression using CIDI-SF through a face-to-face interview by trained health professionals. Participants who reported at least 3 out of 7 depression symptoms (i.e., 1) weight change, 2) difficulty in sleeping, 3) losing interest in things, 4) feeling tired or low on energy, 5) trouble concentrating, 6) feeling worthless, or 7) thoughts about death) in the CIDI-SF questionnaire were considered likely to have major depression [33].

### Covariates

Adjustment for covariates was performed in this analysis, including sociodemographic characteristics (i.e., age, gender, marital status, education level and annual household income), lifestyle habits (i.e., smoking status, alcohol assumption, and physical activity), health status (i.e., BMI and blood pressure), stressful life events in the past two years, passive smoking, cookstove ventilation, and exposure to solid fuels used for heating. Smoking status was classified into four categories: 1) never smoke, 2) quitted, 3) occasional smoker, and 4) current smoker. Participants were classified as a “regular alcohol drinker” if they reported that they drank alcohol “usually at least once a week.” Otherwise, they were classified as a “non-regular drinker.” Physical activity was estimated as metabolic equivalent task hours per day spent on activities related to occupation, commuting, housework, and non-sedentary leisure-time activities. Exposure to stressful life events (Yes/No) was defined as the occurrence of common major life events in the past two years, such as death of a spouse, marital separation/divorce, traffic accident and major natural disaster. Exposure to passive smoking was assessed by self-report responses to the question related to frequency of secondhand smoking exposure. The variable was categorized into 4 levels (none, > 0 to 2 h/week, > 2 to 12 h/week, > 12 h/week). The cut-off points were conventionally selected based on the tertile points among those who had exposure to passive smoking, with the three exposure categories being anticipated to reflect low, middle and high levels of exposure to passive smoking.

### Statistical analysis

All statistical analyses were conducted using the IBM SPSS 25.0 (IBM Corp., Armonk, NY). Data were summarized descriptively using statistics including means, standard deviations, frequencies and percentages. For continuous variables, skewness statistics and normality probability plots were used to assess normality. In this study, the outcome of interest was status of major depressive episode in the past year (Yes/No). The primary exposure of interest was duration of solid fuels used for cooking which was categorized into four levels. Specifically, those participants who had no previous exposure to solid fuels used for cooking or always used clean fuels were categorized as the reference group. The remaining participants were conventionally stratified into three tertiles to characterize low, middle and high levels of exposure with totally four levels for the exposure factor: (i) none, (ii) > 0 to 20 years, (iii) > 20 to ≤ 35 years, (iv) > 35 years. Likewise, the exposure to solid fuels used for heating was categorized into four levels: (i) none, (ii) > 0 to 8.2 years, (iii) > 8.2 to ≤ 13.5 years, (iv) > 13.5 years. The association between major depression in the past year and exposure to solid fuels used for cooking was examined by logistic regression analysis. Unadjusted and adjusted logistic regression analyses were conducted with adjustment for the covariates of sociodemographic characteristics and lifestyle habits, presence of stressful life events in the past two years, presence of cookstove ventilation, exposure to passive smoking, and level of exposure to solid fuels used for heating. As the time scope of the outcome of major depressive episode was the past 12 months from the time of survey, it was possible that some participants might have a major depressive episode prior to exposure to solid fuel usage. A sensitivity analysis was therefore conducted by excluding those participants who had no more than one year of solid fuel usage before the survey. All tests involved were 2-sided at 5% level of significance.

A total of 283,170 participants were included in this secondary data analysis study. Among them, 2,171 participants were classified as having major depressive episode in the past year, and there were totally 91,611 participants without exposure to solid fuels used for cooking and 61,873 to 65,612 participants with different levels of exposure to solid fuels used for cooking. Such a sample size is adequate to detect an odds ratio of having major depressive episode of as small as 1.17 when comparing anyone of the exposure groups with the non-exposure group with over 80% power at 2-sided 5% level of significance.

## Results

### Characteristics of the study population

Amongst 283,170 participants who were included in the baseline survey of the CKB study, the average age was 51.4 (SD = 10.5) years, and 58.2% of them were female. About 68% of them used solid fuels for cooking, with a 27-year median. More than half of the study sample (67%) had at least some cookstove ventilation. Nearly 23% participants had exposure to passive smoking for more than 12 h per week. A total of 2,171 (0.8%) participants reported major depressive episode in the past year. Characteristics of the study population stratified by levels of exposure to solid fuels used for cooking are shown in Table 1.

**Table 1:** Characteristics of the study population by level of exposure to solid fuels used for cooking (*N* = 283,170)

		**Level of exposure to solid fuels used for cooking**			
***Characteristics***	All (*N* = 283,170)	None (*n* = 91,611)	> 0 to 20 years (*n* = 64,524)	> 20 to 35 years (*n* = 65,162)	> 35 years (*n* = 61,873)
***Demographics***					
Age (years)					
30 – < 40	46,577 (16.4%)	15,809 (17.3%)	20,298 (31.5%)	5053 (7.8%)	5417 (8.8%)
40 – < 50	84,487 (29.8%)	27,860 (30.4%)	21,798 (33.8%)	23,623 (36.3%)	11,206 (18.1%)
50 – < 60	88,697 (31.3%)	27,153 (29.6%)	13,257 (20.5%)	28,678 (44.0%)	19,609 (31.7%)
60 – < 70	48,441 (17.1%)	15,640 (17.1%)	6924 (10.7%)	6181 (9.5%)	19,696 (31.8%)
≥ 70	14,968 (5.3%)	5149 (5.6%)	2247 (3.5%)	1627 (2.5%)	5945 (9.6%)
Sex					
Male	118,260 (41.8%)	79,634 (86.9%)	20,576 (31.9%)	9111 (14.0%)	8939 (14.4%)
Female	164,910 (58.2%)	11,977 (13.1%)	43,948 (68.1%)	56,051 (86.0%)	52,934 (85.6%)
Marital status					
Married	259,280 (91.6%)	87,231 (95.2%)	60,204 (93.3%)	60,226 (92.4%)	51,619 (83.4%)
Widowed / separated / divorced	21,671 (7.7%)	3666 (4.0%)	3906 (6.1%)	4551 (7.0%)	9548 (15.4%)
Never married	2219 (0.8%)	714 (0.8%)	414 (0.6%)	385 (0.6%)	706 (1.1%)
Highest education attainment					
No formal school	67,740 (23.9%)	13,190 (14.4%)	12,506 (19.4%)	18,147 (27.8%)	23,897 (38.6%)
Primary school	118,593 (41.9%)	37,707 (41.2%)	24,332 (37.7%)	28,739 (44.1%)	27,815 (45.0%)
Middle school / high school	93,744 (33.1%)	38,923 (42.5%)	26,722 (41.4%)	18,032 (27.7%)	10,067 (16.3%)
Technical school / college/ university	3093 (1.1%)	1791 (2.0%)	964 (1.5%)	244 (0.4%)	94 (0.2%)
Household income in last year (Yuan)					
< 5,000	41,918 (14.8%)	10,238 (11.2%)	6634 (10.3%)	8255 (12.7%)	16,791 (27.1%)
5,000 – 9,999	70,752 (25.0%)	20,794 (22.7%)	14,517 (22.5%)	16,663 (25.6%)	18,778 (30.3%)
10,000 – 19,999	81,352 (28.7%)	25,492 (27.8%)	19,283 (29.9%)	20,737 (31.8%)	15,840 (25.6%)
20,000 – 34,999	54,285 (19.2%)	19,522 (21.3%)	14,783 (22.9%)	12,725 (19.5%)	7255 (11.7%)
≥ 35,000	34,863 (12.3%)	15,565 (17.0%)	9307 (14.4%)	6782 (10.4%)	3209 (5.2%)
***Obesity status and lifestyle characteristics***					
Obesity status					
Normal weight (18.5 ≤ BMI < 23.9)	162,222 (57.3%)	56,475 (61.6%)	36,709 (56.9%)	34,507 (53.0%)	34,531 (55.8%)
Overweight (24.0 ≤ BMI < 27.9)	82,596 (29.2%)	24,858 (27.1%)	19,271 (29.9%)	20,821 (32.0%)	17,646 (28.5%)
Obese (BMI ≥ 28.0)	22,982 (8.1%)	5442 (5.9%)	5379 (8.3%)	6813 (10.5%)	5348 (8.6%)
Under weight (BMI < 18.5)	15,368 (5.4%)	4835 (5.3%)	3165 (4.9%)	3021 (4.6%)	4347 (7.0%)
Smoking status					
Never smoke	175,263 (61.9%)	23,540 (25.7%)	46,351 (71.8%)	55,238 (84.8%)	50,134 (81.0%)
Quitted	16,919 (6.0%)	9841 (10.7%)	3181 (4.9%)	1614 (2.5%)	2283 (3.7%)
Occasional smoker	12,954 (4.6%)	7445 (8.1%)	2438 (3.8%)	1455 (2.2%)	1616 (2.6%)
Current smoker	78,034 (27.6%)	50,785 (55.4%)	12,554 (19.5%)	6855 (10.5%)	7840 (12.7%)
Regular alcohol drinker					
No	245,061 (86.5%)	69,241 (75.6%)	57,499 (89.1%)	60,967 (93.6%)	57,354 (92.7%)
Yes	38,109 (13.5%)	22,370 (24.4%)	7025 (10.9%)	4195 (6.4%)	4519 (7.3%)
Physical activity, MET – hours/day^a^	23.2 (14.5)	24.8 (16.2)	24.4 (14.7)	22.2 (13.0)	20.7 (12.4)
***Stressful life event & sleep disturbance***					
Stressful life event in the past two years					
No	261,388 (92.3%)	85,582 (93.4%)	59,660 (92.5%)	59,988 (92.1%)	56,158 (90.8%)
Yes	21,782 (7.7%)	6029 (6.6%)	4864 (7.5%)	5174 (7.9%)	5715 (9.2%)
***Cook stove ventilation & passive smoking exposure***					
Had at least some cook stove ventilation					
No	94,242 (33.3%)	27,318 (29.9%)	23,802 (36.9%)	24,207 (37.2%)	18,915 (30.6%)
Yes	188,570 (66.7%)	64,034 (70.1%)	40,661 (63.1%)	40,934 (62.8%)	42,941 (69.4%)
Passive smoking exposure					
None	91,358 (32.3%)	28,650 (31.3%)	22,290 (34.5%)	19,727 (30.3%)	20,691 (33.4%)
> 0 to 2 h/week	62,116 (21.9%)	18,898 (20.6%)	14,138 (21.9%)	15,068 (23.1%)	14,012 (22.6%)
> 2 to 12 h/week	65,490 (23.1%)	22,473 (24.5%)	14,402 (22.3%)	14,921 (22.9%)	13,694 (22.1%)
> 12 h/week	64,206 (22.7%)	21,590 (23.6%)	13,694 (21.2%)	15,446 (23.7%)	13,476 (21.8%)
***Solid fuels usage for heating***					
Level of exposure to solid fuels used for heating^b^					
None	108,153 (38.5%)	38,143 (42.0%)	26,444 (41.3%)	22,064 (34.1%)	21,502 (35.1%)
> 0 to 8.2 years	57,408 (20.4%)	13,163 (14.5%)	18,968 (29.6%)	16,993 (26.3%)	8284 (13.5%)
> 8.2 to 13.5 years	59,153 (21.1%)	19,568 (21.5%)	11,307 (17.6%)	14,500 (22.4%)	13,778 (22.5%)
> 13.5 years	56,252 (20.0%)	19,975 (22.0%)	7385 (11.5%)	11,155 (17.2%)	17,737 (28.9%)
***Major depression***					
Major depression episode in the past year					
No	280,999 (99.2%)	91,161 (99.5%)	64,032 (99.2%)	64,548 (99.1%)	61,258 (99.0%)
Yes	2171 (0.8%)	450 (0.5%)	492 (0.8%)	614 (0.9%)	615 (1.0%)

Variables with data marked with ^a^ are presented as mean (standard deviation), all others are presented as frequency (%)

^b^There were less than 0.8% (*n* = 2204) of participants without detailed information about fuels used for heating

### Association between household use of solid fuels for cooking and major depressive episode

Based on their duration of exposure to solid fuels used for cooking, participants were categorized into four levels: (i) none, (ii) > 0 to 20 years, (iii) > 20 to ≤ 35 years, (iv) > 35 years. Those participants who had no previous exposure to solid fuels used for cooking or always used clean fuels for cooking were categorized as the reference group (none exposure). The remaining participants were conventionally stratified into three tertiles to characterize low, middle and high levels of exposure. Unadjusted logistic regression analysis showed that an increased level of exposure to solid fuels used for cooking was associated with an increased odds of having a major depressive episode (unadjusted model in Table 2). After adjusting for sociodemographic characteristics, obesity and lifestyle habits, presence of stressful life events, presence of cookstove ventilation, passive smoking exposure, and level of exposure to solid fuels used for heating, the pattern of association between an increased odds of having a major depressive episode and an increased level of exposure was also noted. Participants who had exposure to solid fuels used for cooking for up to 20 years, more than 20 to 35 years, and more than 35 years were 1.09 (95% CI 0.94–1.27), 1.18 (95% CI: 1.01–1.38) and 1.19 (95% CI: 1.01–1.40) times greater odds of having a major depressive episode, respectively, compared with those who had no previous exposure to solid fuel used for cooking or always used clean fuels for cooking (adjusted model 1 in Table 2). A sensitivity analysis was conducted by excluding those participants who had no more than one year of solid fuel usage before the survey, the results were similar to the primary analysis one (adjusted model 2 in Table 2).

**Table 2:** Risk of major depression episode by level of exposure to solid fuels used for cooking

	Sensitivity analysis					
	Unadjusted model		Adjusted model 1		Adjusted model 2	
**Exposure factor**	Odds ratio (95% CI)	*p*-value	Odds ratio (95% CI)	*p*-value	Odds ratio (95% CI)	*p*-value
*Level of exposure to solid fuels used for cooking*						
None (ref)	1		1		1	
> 0 to 20 years	1.56 (1.37 – 1.77)	< 0.001	1.09 (0.94 – 1.27)	0.249	1.08 (0.93 – 1.25)	0.316
> 20 to 35 years	1.93 (1.71 – 2.18)	< 0.001	1.18 (1.01 – 1.38)	0.034	1.18 (1.01 – 1.38)	0.041
> 35 years	2.03 (1.80 – 2.30)	< 0.001	1.19 (1.01 – 1.40)	0.033	1.18 (1.01 – 1.39)	0.043

Model 1: with adjustment for demographic, obesity and lifestyle characteristics, presence of stressful life event, presence of cook stove ventilation, passive smoking exposure, and level of exposure to solid fuels used for heating as listed in Table 1

Model 2: with adjustment for covariates in model 1 and excluding those participants who used solid fuels for cooking but had no more than one year of solid fuel used for cooking into analysis (*n* = 622 excluded)

*ref* reference category for calculating odds ratios of other comparison categories

## Discussion

Approximately 46% of the population in China used solid fuels as a household energy source, leading to household air pollution; and the proportion was substantially higher in rural areas [13, 23]. In fact, the present study found that 68% of rural residents used solid fuels for cooking. To the best of our knowledge, this is the largest national study to explore the relationship between solid fuel use and depression in rural China. The results revealed an association between household use of solid fuels for cooking and major depression, particularly for those who had used solid fuels for more than 20 years, after controlling for potential confounding covariates, including sociodemographic characteristics, lifestyle habits, health status, presence of stressful life events, presence of cookstove ventilation, passive smoking exposure, and exposure to solid fuels used for heating. Although participants with longer exposure generally associated with an increased odds of having a major depressive episode, the odds ratio of the longest exposure group (> 35 years, OR = 1.19) was unexpectedly similar to the second longest exposure group (> 20 to 35 years, OR = 1.18). A possible explanation may be owing to the fact that people with longer exposure were more likely subject to a competing risk of death, which may diminish the strength of association, particularly in the longest exposure group.

There is a growing body of evidence that solid fuel use is associated with a high risk of depression [22, 23], which is consistent with the current findings. Individuals (*N*= 8637) with exposure to solid fuel combustion for over 4 years had 1.12 times greater odds of having depressive symptoms [23]. Supported by the following longitudinal survey (*N*= 7005) [22], individuals using solid fuels in cooking for more than 7 years had 1.36 times greater odds of depression risk than those who always used clean fuels.

This study, together with the aforementioned previous studies, provides evidence on the association between the exposure to solid fuels and the prevalence of depression. However, only limited evidence exists on the mechanisms linking the use of solid fuels for cooking with depression. The incomplete combustion of solid fuels generates various air pollutants including PM, carbon monoxide, sulfur oxides, and polycyclic aromatic hydrocarbons [34, 35]. One possible explanation may be that inhalation of air pollutants can trigger associated oxidative stress, cerebrovascular damage, neuroinflammation, and neurodegenerative pathology, which all might cause or exacerbate the risk of depression [36–38]. Animal experience revealed that PM might cause neurotoxicity by inducing microglia activation characterized by the release of TNFα, which damages the olfactory bulb and increases depression risk [39]. Moreover, studies indicated that PM causes elevated levels of cortisol [40], which has been related to the development of depression [41]. Furthermore, domestic cooking with solid fuels could increase the risk of chronic diseases, such as cancer and cardiorespiratory diseases [19, 42], which are strongly associated with depression [43, 44].

In rural China, solid fuels are reported to be the dominant cooking fuel, with biomass and coal accounting for 47.6% [45] and 13.5% [46], respectively. Our study gives valuable insights into the potential hazardous effects of using solid fuels for cooking on mental health. It indicates household solid fuels used for cooking is a critical public health issue and that policy makers must take responsibility to make the needed policy changes. It is necessary to encourage people to switch to cleaner fuels and technologies when cooking to reduce exposure to household air pollution. Moreover, in this study, depressive episode was more prevalent in those without cookstove ventilation. This result is in line with those of the previous studies [22, 47], showing that cooking ventilation may weaken the relationship of cooking with solid fuel and long duration cooking with depressive symptoms, suggesting that improvements in cooking ventilation should be strongly encouraged.

As a remark, although people with longer exposure to solid fuels used for cooking generally associated with an increased odds of having a major depression episode, the odds ratio of the longest exposure group (> 35 years, OR = 1.19) was unexpectedly similar to the second longest exposure group (> 20 to 35 years, OR = 1.18). A possible explanation may be owing to the fact that people with longer exposure were more likely subject to a competing risk of death, which may diminish the strength of association, particularly in the longest exposure group.

Despite the significance of the findings, there are several limitations in this study that may impact the generalisability of this study. First, the cross-sectional study design assesses both outcome of interest and exposure simultaneously. Therefore, it may not be able to establish a cause-and-effect relationship between household solid fuels used for cooking and depression. In addition, self-reported information is prone to recall bias when participants fail to accurately remember an event in the past. Nonetheless, the overestimation or underestimation of association between cause and effect may be resolved through a longitudinal cohort study in which an event may be observed first, followed by the effects. On the other hand, different cooking practices and chemical properties of fuel such as density, volatility and thermal capacity which could affect the indoor air pollution were not examined in the CKB study. Hence, this could result in imprecision of actual exposure to solid fuels used for cooking. Although this study had controlled for potential confounders (e.g., sociodemographic characteristics, obesity status and lifestyle habits, presence of stressful life events, presence of cookstove ventilation and passive smoking exposure), the results might be confounded by other unmeasured covariates. This is because our study was a secondary data analysis where the adjusted analysis was only able to be performed based on existing available variables.

## Conclusion

This study demonstrates the significant association between the use of household solid fuels for cooking and the prevalence of depression in rural China; and the longer duration of exposure, the higher odds of having a depressive episode. Further studies are warranted to examine if there is a causal relationship between them. Nevertheless, reducing the use of solid fuels for cooking by promoting the use of clean energy should be encouraged.

## Data Availability

The datasets used and analyzed during this current study are available from the corresponding author on reasonable request.
